# Supplementary material for: Arbuscular Mycorrhizal Symbiosis Primes Tolerance to Cucumber Mosaic Virus in Tomato
Source: Viruses. 2020 Jun 22;12(6):675. doi: 10.3390/v12060675 (PMC7354615; doi:10.3390/v12060675)
Supplement: Supplementary file 1 [file viruses-12-00675-s001.zip › FigureS1.pdf]

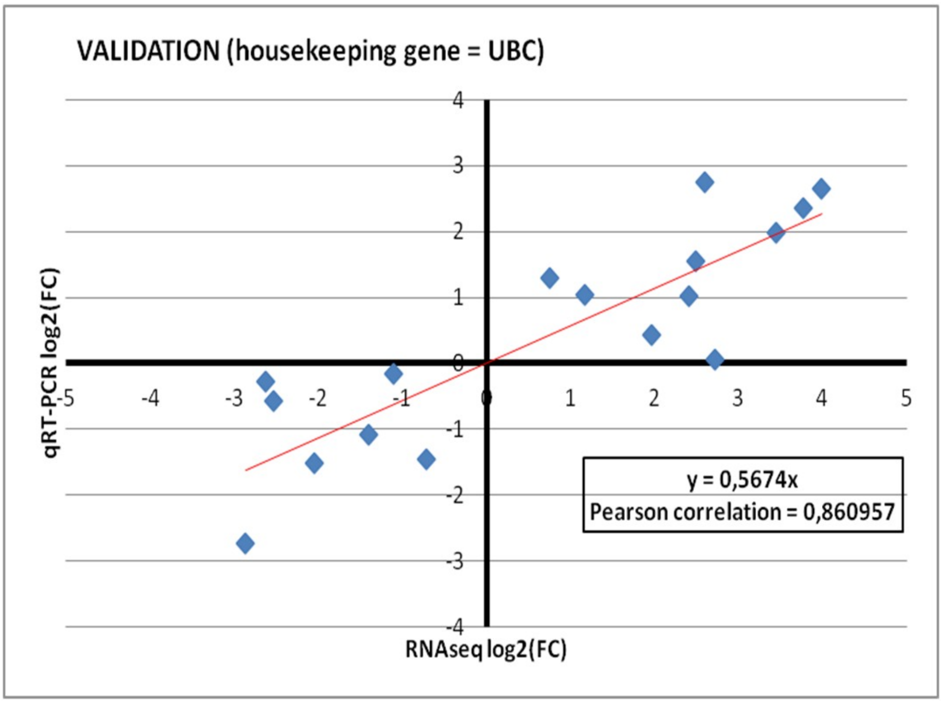


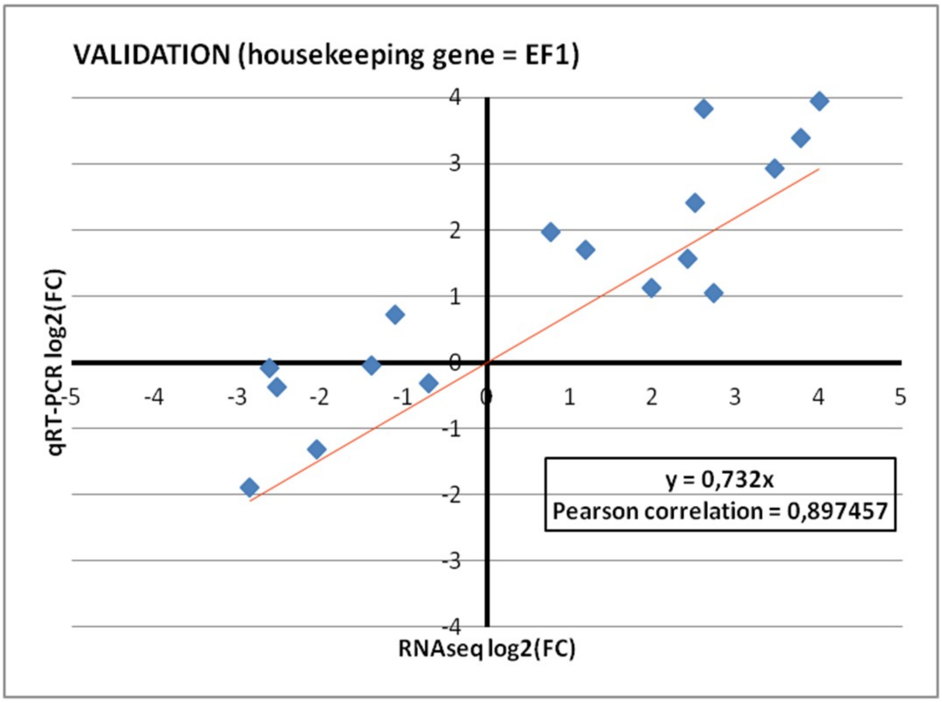


**Figure S1.** Correlation between expression values measured by RNAseq and qRT-PCR. Expression values are reported as the log2 of Fold Change (FC) in respect to C plants.
