# Supplementary material for: Arbuscular Mycorrhizal Symbiosis Primes Tolerance to Cucumber Mosaic Virus in Tomato
Source: Viruses. 2020 Jun 22;12(6):675. doi: 10.3390/v12060675 (PMC7354615; doi:10.3390/v12060675)
Supplement: Supplementary file 1 [file viruses-12-00675-s001.zip › FigureS2.pdf]

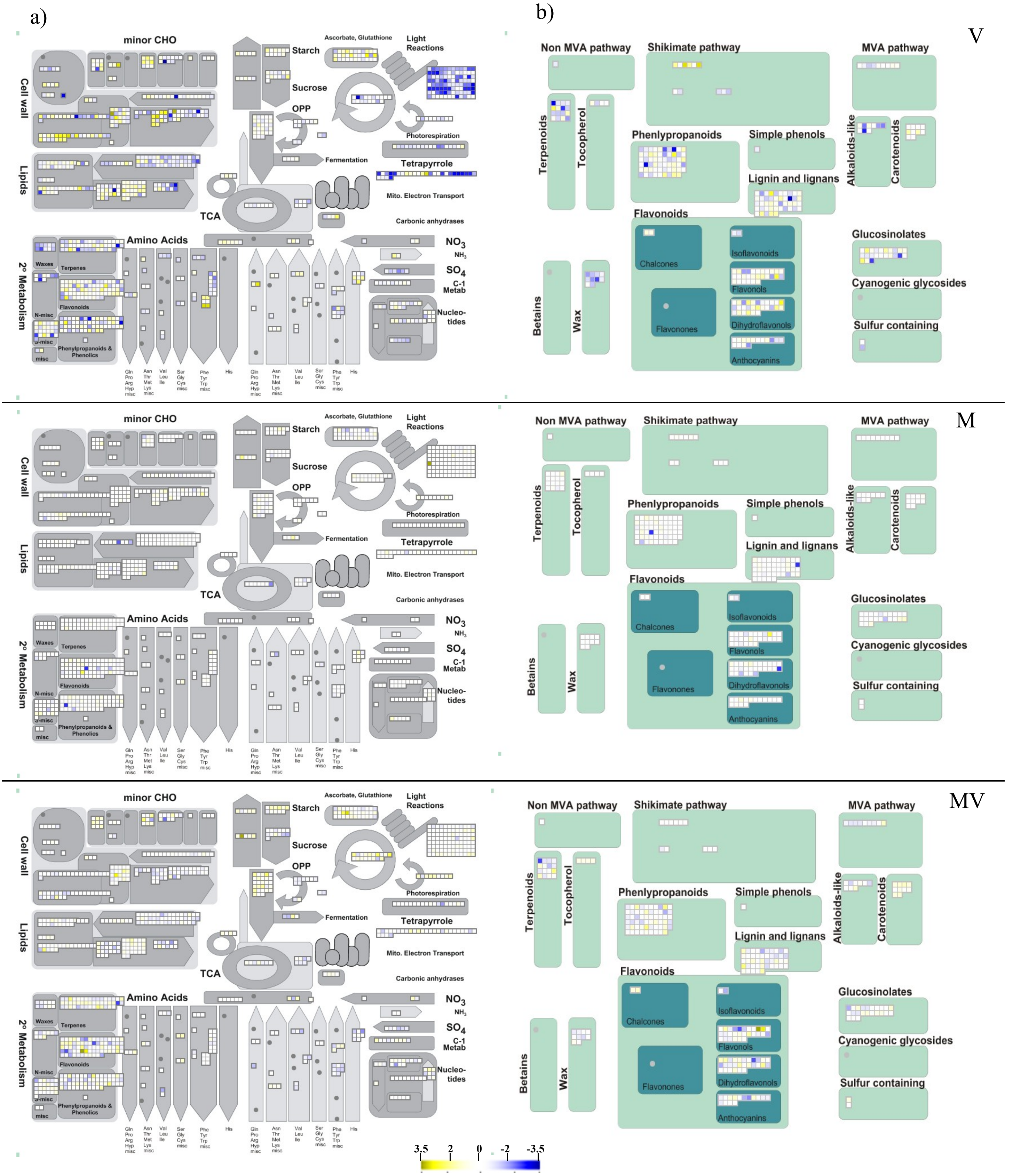
a)
b)
V
M
MV
minor CHO
Cell wall
Lipids
Starch
Sucrose
OPP
Ascorbate, Glutathione
Light Reactions
Photorespiration
Tetrapyrrole
Fermentation
TCA
Mito. Electron Transport
Carbonic anhydrases
Amino Acids
NO3
NH3
SO4
C-1 Metab
Nucleo-tides
2° Metabolism
Waxes
Terpenes
Flavonoids
N-misc
S-misc
misc
Phenylpropanoids & Phenolics
Gln Pro Arg Hyp misc
Asn Thr Met Lys misc
Val Leu Ile
Ser Gly Cys misc
Phe Tyr Trp misc
His
Non MVA pathway
Shikimate pathway
MVA pathway
Terpenoids
Tocopherol
Phenlypropanoids
Simple phenols
Lignin and lignans
Alkaloids-like
Carotenoids
Flavonoids
Chalcones
Isoflavonoids
Flavonols
Dihydroflavonols
Flavonones
Anthocyanins
Betains
Wax
Glucosinolates
Cyanogenic glycosides
Sulfur containing
3.5
2
0
-2
-3.5

c)

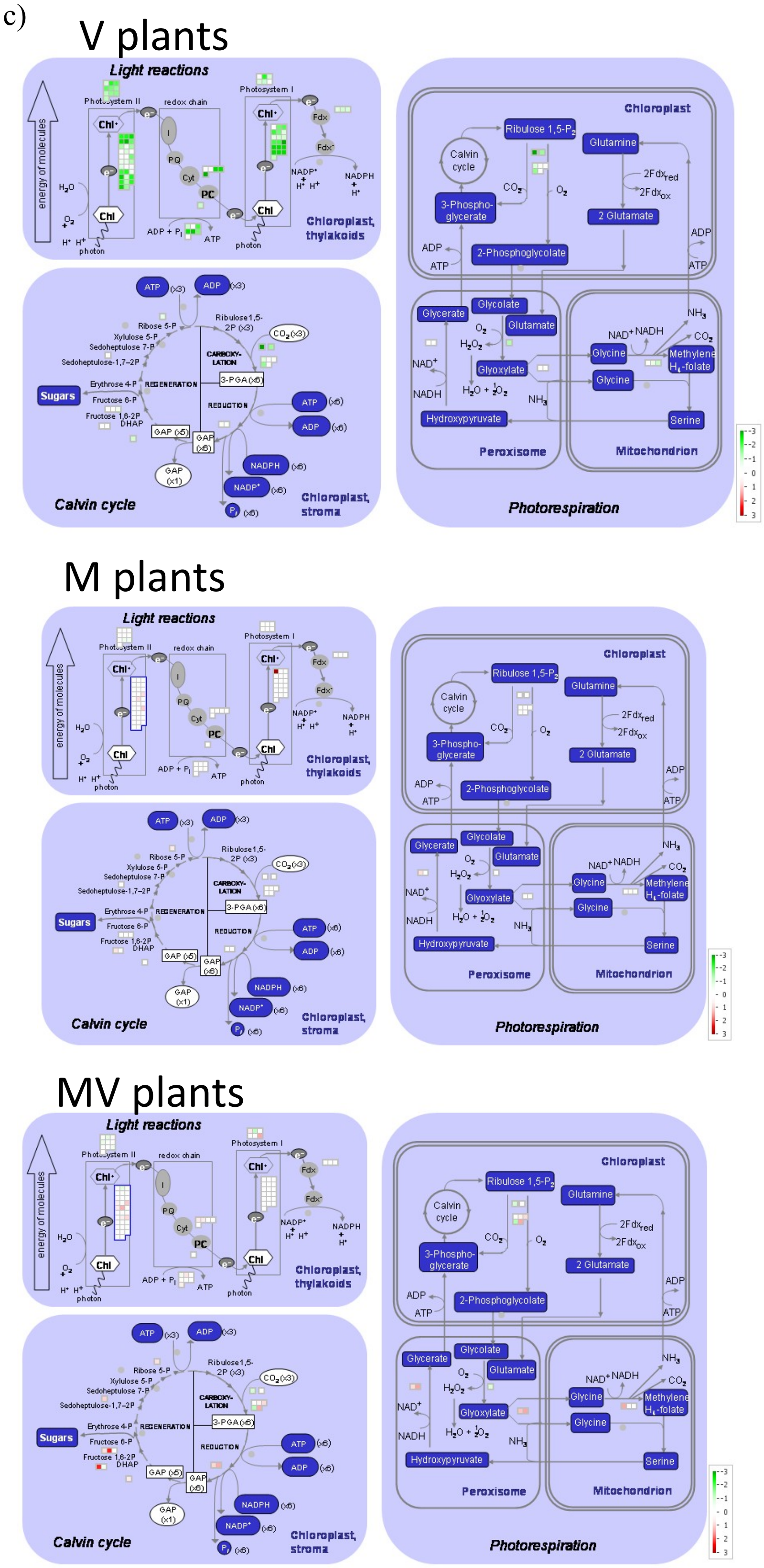

Figure S2. MapMan overview showing differences in transcript levels of DEGs related to (a) primary and (b) secondary metabolism and to photosynthesis (c) in virus-infected (V), mycorrhizal (M) and virus-infected mycorrhizal (MV) plants with respect to control (C) plants. Expression values are reported as the log2 of Fold Change with respect to C plants; yellow and blue colors indicate up- and down-regulation, respectively; according to the legend reported in the figure.
