# Supplementary material for: Arbuscular Mycorrhizal Symbiosis Primes Tolerance to Cucumber Mosaic Virus in Tomato
Source: Viruses. 2020 Jun 22;12(6):675. doi: 10.3390/v12060675 (PMC7354615; doi:10.3390/v12060675)
Supplement: Supplementary file 1 [file viruses-12-00675-s001.zip › FigureS3.pdf]

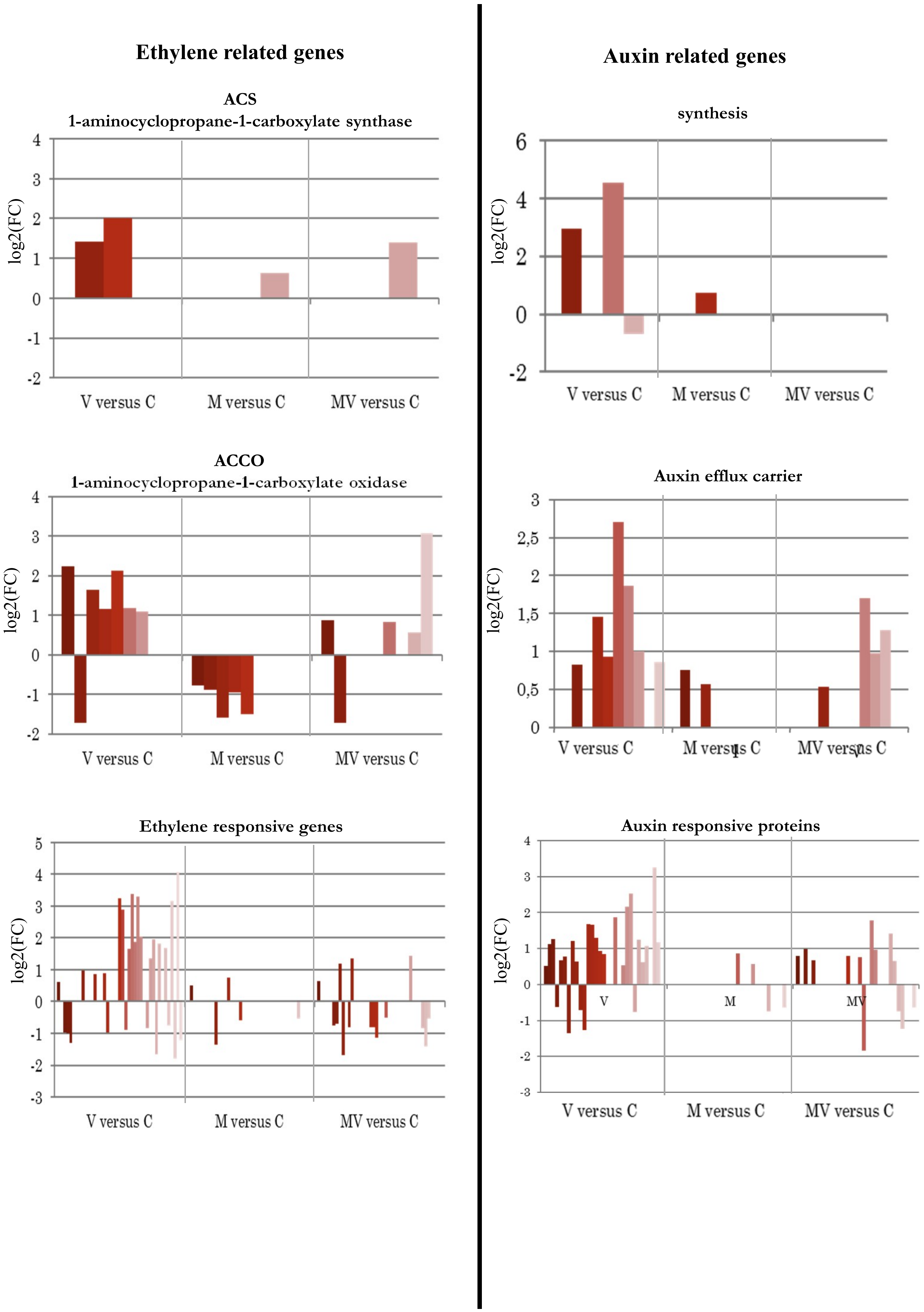

Ethylene related genes
ACS
1-aminocyclopropane-1-carboxylate synthase
log2(FC)
4
3
2
1
0
-1
-2
V versus C
M versus C
MV versus C
ACCO
1-aminocyclopropane-1-carboxylate oxidase
log2(FC)
4
3
2
1
0
-1
-2
V versus C
M versus C
MV versus C
Ethylene responsive genes
log2(FC)
5
4
3
2
1
0
-1
-2
-3
V versus C
M versus C
MV versus C
Auxin related genes
synthesis
log2(FC)
6
4
2
0
-2
V versus C
M versus C
MV versus C
Auxin efflux carrier
log2(FC)
3
2,5
2
1,5
1
0,5
0
V versus C
M versus C
MV versus C
Auxin responsive proteins
log2(FC)
4
3
2
1
0
-1
-2
-3
V
M
MV
V versus C
M versus C
MV versus C

Jasmonate related genes

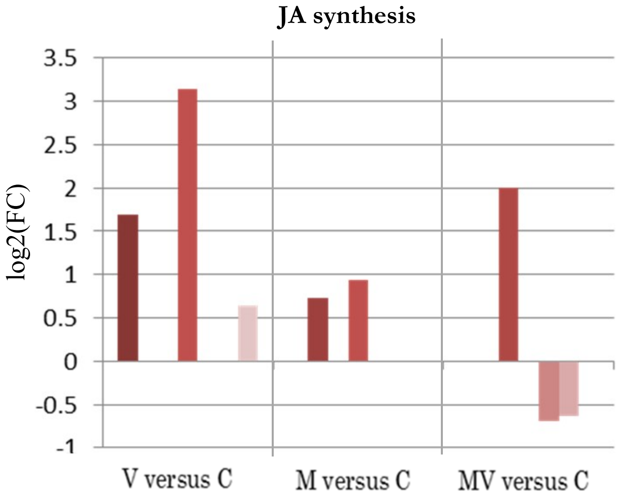

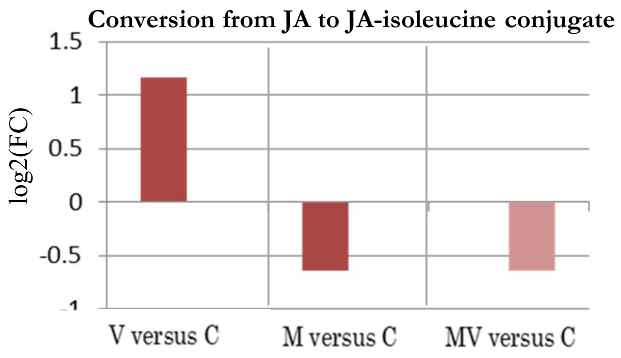

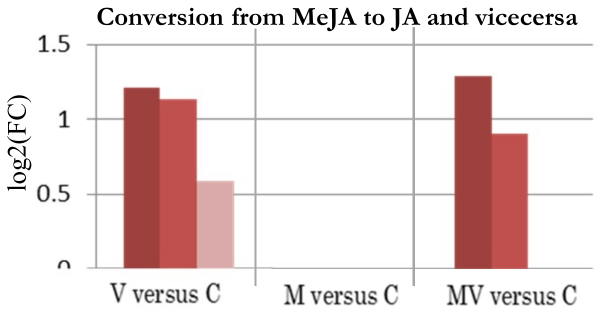

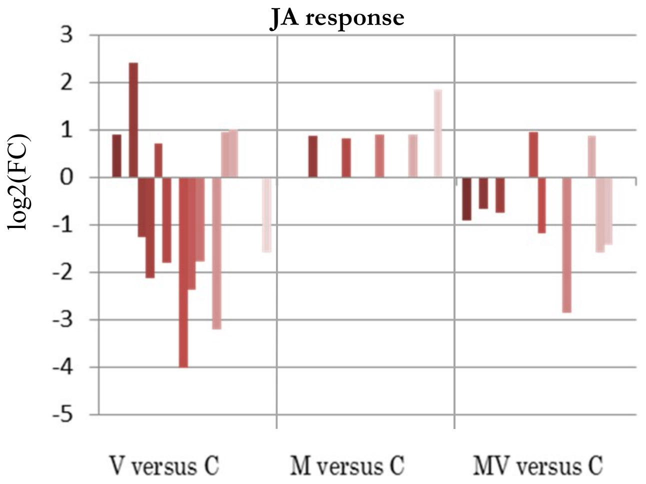

Salicylic acid related genes

SA synthesis

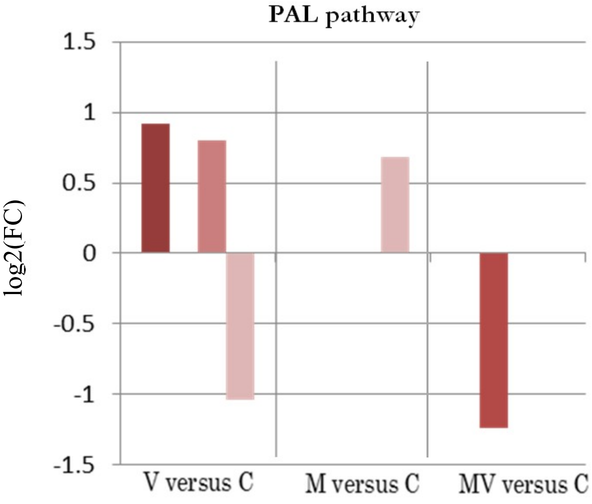

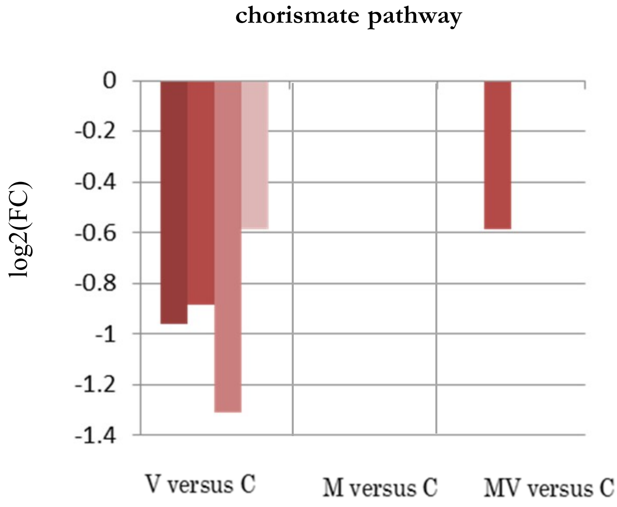

SA – MeSA conversion

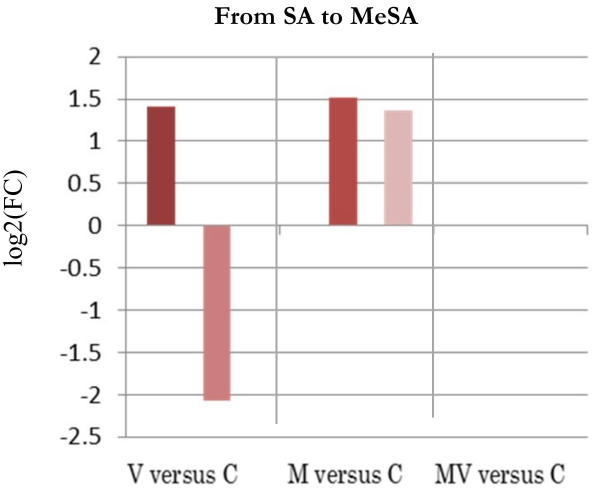

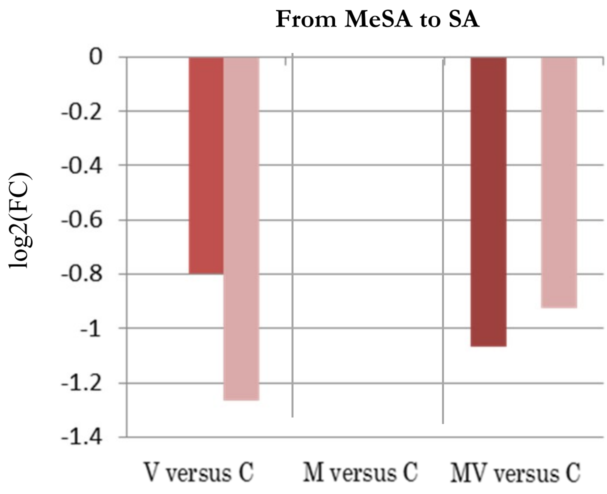

SA response

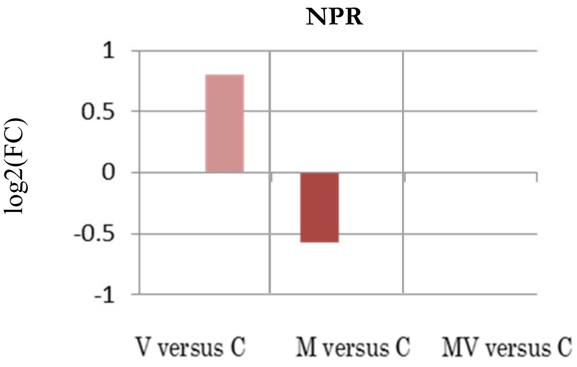

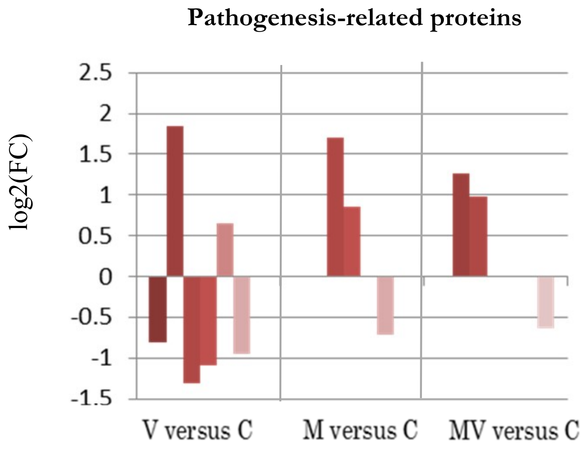

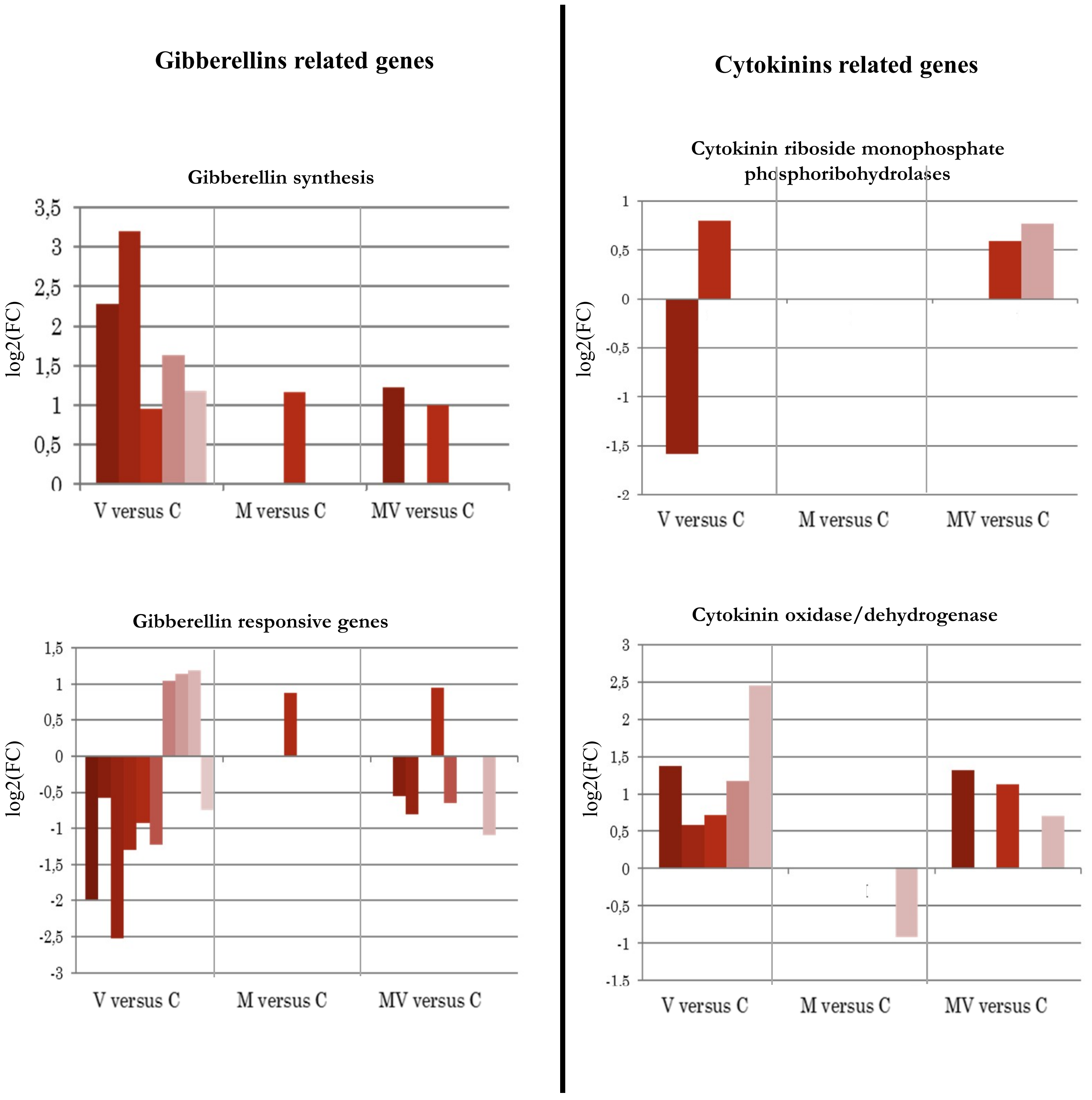

Figure S3. Expression of DEGs related to hormones-related functional categories in virus-infected (V), mycorrhizal (M) and virus-infected mycorrhizal (MV) plants with respect to control (C) plants. Expression values are reported as the log2 of Fold Change (FC) in respect to control (C) plants.
