# Supplementary material for: Arbuscular Mycorrhizal Symbiosis Primes Tolerance to Cucumber Mosaic Virus in Tomato
Source: Viruses. 2020 Jun 22;12(6):675. doi: 10.3390/v12060675 (PMC7354615; doi:10.3390/v12060675)
Supplement: Supplementary file 1 [file viruses-12-00675-s001.zip › FigureS4.pdf]

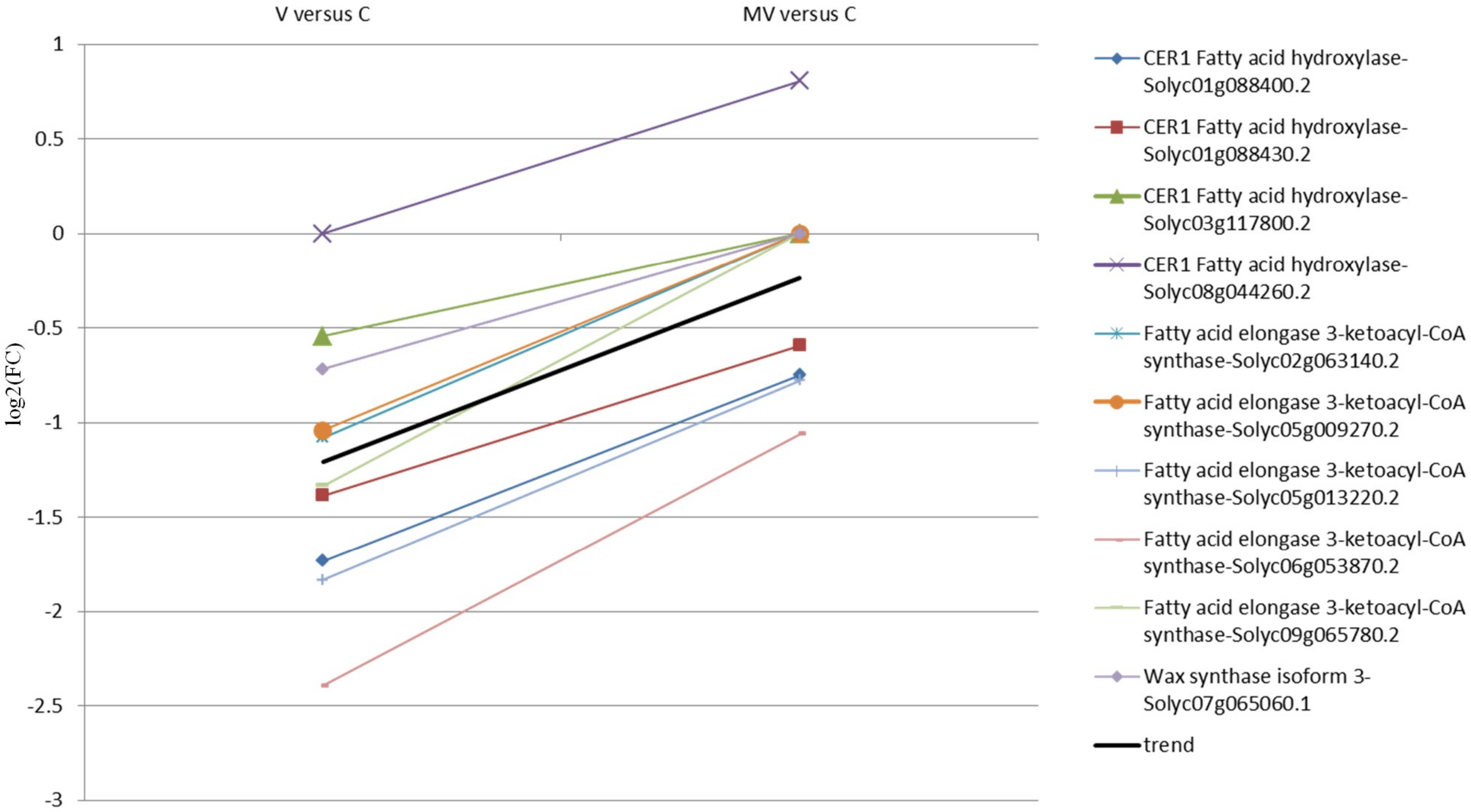

Figure S4. Regulation of DEGs involved in cuticular waxes synthesis in CMV-infected (V) and CMV-infected mycorrhizal plants (MV) plants in respect to control (C) plants. Expression values are reported as the log2 of Fold Change (FC) in respect to C plants. Black line indicates the regulation trend.
