# Supplementary material for: Arbuscular Mycorrhizal Symbiosis Primes Tolerance to Cucumber Mosaic Virus in Tomato
Source: Viruses. 2020 Jun 22;12(6):675. doi: 10.3390/v12060675 (PMC7354615; doi:10.3390/v12060675)
Supplement: Supplementary file 1 [file viruses-12-00675-s001.zip › FigureS5.pdf]

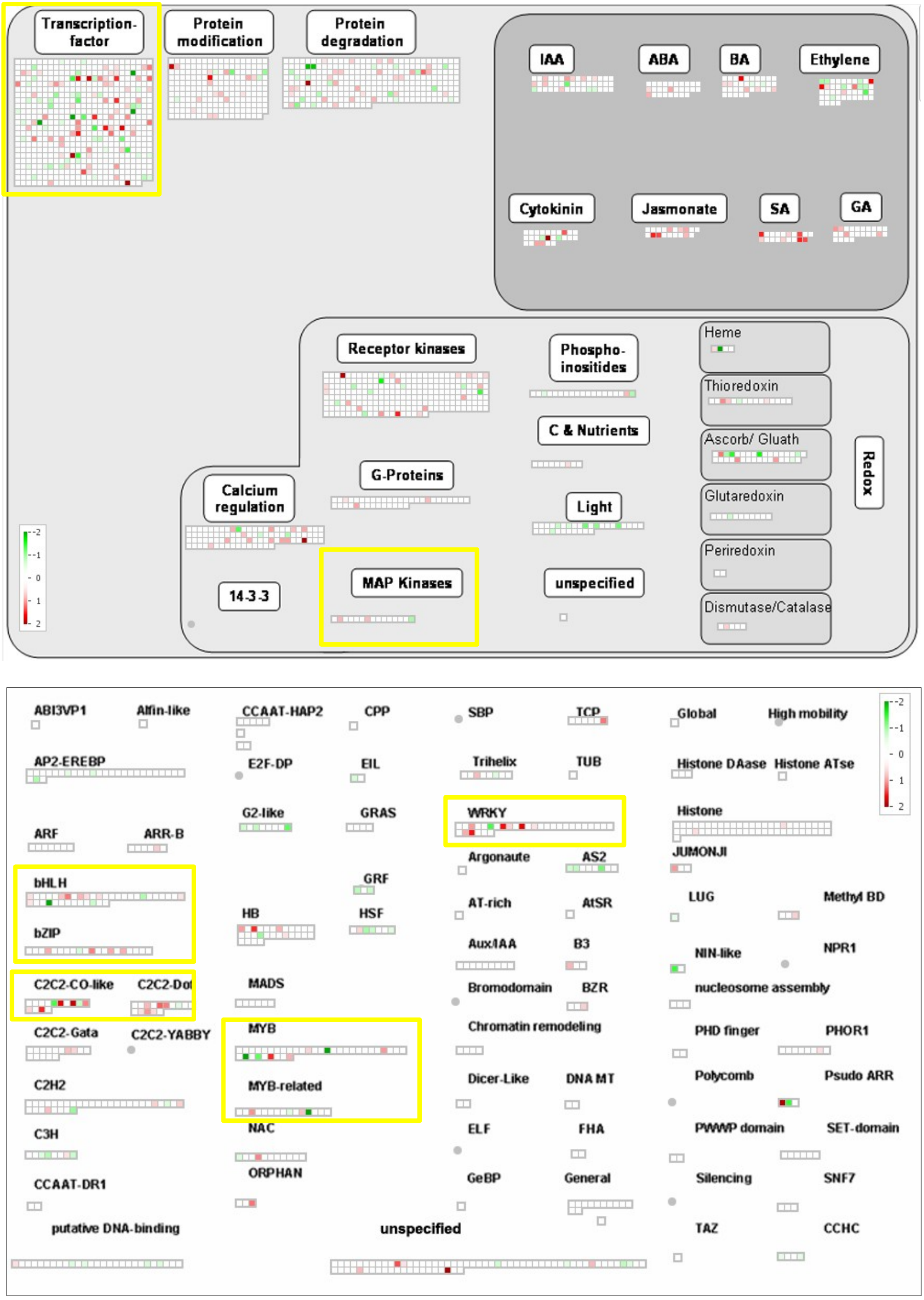

Figure S5. MapMan visualization of expression of transcription factor families in mycorrhizal (M) plants with respect to control (C) plants. Expression values are reported as the log2 of Fold Change (FC) in respect to control (C) plants
