# Supplementary material for: Arbuscular Mycorrhizal Symbiosis Primes Tolerance to Cucumber Mosaic Virus in Tomato
Source: Viruses. 2020 Jun 22;12(6):675. doi: 10.3390/v12060675 (PMC7354615; doi:10.3390/v12060675)
Supplement: Supplementary file 1 [file viruses-12-00675-s001.zip › TableS1.pdf]

Gene ID	Description	primer forward 5'-3'	primer reverse 5'-3'
**CMV_CP**	CMV coat protein	TTATCCGTTGCCGCCATCTC	TGTTGTTGGCTTGGACTCCA
**Solyc06g072570.1.1**	Ubiquitin Conjugating Enzyme	ACCTGATGATCCACAAGATGC	GCAGCAAGTGTGGATGTTTTT
**Solyc12g100330.1.1**	chromomethylase 3	GTCAAATTCCAAAGCGGAAG	CGAAGCTCATCGCATAGTCA
**Solyc 10g052470.1.1**	Myb family transcription factor (fragment)	CTTCTGGATCATGGACTGCGA	CTGCTGTTTTGCCTCCAACTG
**Solyc02g094520.2.1**	Histone-lysine N-methyltransferase	GCCTGATCTAAAGGCGATTTC	GCGAAAAGAACTGATGACCAA
**Solyc 07g008240.2.1**	Non-symbiotic hemoglobin protein	TGGAAAGGTTGTGGTGAGGG	AGTGCTCATCAACCACACCA
**Solyc 01g008150.1.1**	Unknown protein (AHRD V1)	TACATGAACGGTGGTGAGCC	CCGCGAAAACTCCATCATCG
**Solyc 04g071800.2.1**	Cytochrome P450	GGCTCATGGCTATTTGCATT	GTGGGAGTGAACCAAGGAGA
**Solyc 10g085870.1.1**	UDP-glucosyltransferase family 1 protein	GGAACAAGGCTGCAATTGAT	ACCATCTGAGACGTGGGAAG
**Solyc 09g092130.2.1**	Sucrose phosphate Synthase	GGATCTTGCAGCACTCACAA	AGGGTCTGGTCTGGACAATG
**Solyc 05g005280.2.1**	Poly polymerase catalytic domain containing protein expressed	GCTTGGTTTGGTTCTTCCAA	CTTCCACTGCCGAAAATACC

Table S1 List of primers used in this study
