# Supplementary material for: Arbuscular Mycorrhizal Symbiosis Primes Tolerance to Cucumber Mosaic Virus in Tomato
Source: Viruses. 2020 Jun 22;12(6):675. doi: 10.3390/v12060675 (PMC7354615; doi:10.3390/v12060675)
Supplement: Supplementary file 1 [file viruses-12-00675-s001.zip › TableS2.pdf]

Table S2 Bioinformatics parameters used in the mRNA-seq data analysis

General hardware settings: 40 CPU and RAM 150 GB.

Parameters settings:

- Tophat: -p 20, --bowtie1, -i30, -I200000, --min-segment-intron 30, --max-segment-intron 200000, --min-coverage-intron 30, --max-coverage-intron 200000, other default parameters
- cufflinks: -p20, --min-intron-length 30, other default parameters
- cuffcompare: -p20, -R, other default parameters
- Cuffmerge: -p20, other default parameters
- Cuffdiff: -p20, -u, -v, other default parameters
