# Supplementary material for: Arbuscular Mycorrhizal Symbiosis Primes Tolerance to Cucumber Mosaic Virus in Tomato
Source: Viruses. 2020 Jun 22;12(6):675. doi: 10.3390/v12060675 (PMC7354615; doi:10.3390/v12060675)
Supplement: Supplementary file 1 [file viruses-12-00675-s001.zip › TableS7.pdf]

Table S7 mRNA-seq read statistics

Samples	Total reads	Mapped read	Transcriptome coverage	Uniquely mapped reads	Multiple mapped reads
C_1	10,552,060	8,660,453 (82.1%)	15x	8,483,613	176,840
C_2	10,117,165	8,187,582 (80.9%)	15x	7,942,025	245,557
C_3	15,221,311	12,708,561 (83.5%)	23x	12,377,330	331,231
V_1	17,436,750	14,785,997 (84.8%)	26x	14,546,406	239,591
V_2	14,596,457	12,384,474 (84.8%)	22x	12,173,062	211,412
V_3	16,310,309	13,854,892 (84.9%)	25x	13,645,742	209,150
M_1	14,373,229	11,815,972 (82.2%)	21x	11,521,651	294,321
M_2	18,210,733	15,218,574 (83.6%)	27x	14,935,259	283,315
M_3	20,234,902	14,220,456 (70.3%)	25x	13,807,572	412,884
MV_1	14,358,409	12,041,203 (83.9%)	22x	11,815,102	226,101
MV_2	15,085,717	12,738,156 (84.4%)	23x	12,529,088	209,068
MV_3	16,975,460	14,294,838 (84.2%)	26x	14,071,320	223,518
