# Supplementary material for: Arbuscular Mycorrhizal Symbiosis Primes Tolerance to Cucumber Mosaic Virus in Tomato
Source: Viruses. 2020 Jun 22;12(6):675. doi: 10.3390/v12060675 (PMC7354615; doi:10.3390/v12060675)
Supplement: Supplementary file 1 [file viruses-12-00675-s001.zip › TableS9.pdf]

a)

Gene ID	Description	RNA-seq			qRT-PCR		
		V	M	MV	V	M	MV
**Solyc10g052470.1.1**	Myb family transcription factor	2.49	-2.62	-	1.55±024	-0.27±0.38	-
**Solyc12g100330.1.1**	chromomethylase 3	-1.39	-	-	-1.08±0.17	-	-
**Solyc02g094520.2.1**	Histone-lysine N-methyltransferase	-0.71	-	-	-1.46±0.10	-	-
**Solyc07g008240.2.1**	Non-symbiotic hemoglobin protein	-	-2.05	-2.86	-	-1.51±0.33	-2.74±0.17
**Solyc04g071800.2.1**	Cytochrome P450	1.18	-2.53	-1.11	1.04±0.30	-0.57±0.36	-0.16±0.22
**Solyc05g005280.2.1**	Poly polymerase catalytic domain containing protein expressed	2.61	2.73	4.00	2.75±0.21	0.05±0.19	2.66±0.15
**Solyc10g085870.1.1**	UDP-glucosyltransferase family 1 protein	0.76	2.42	3.78	1.29±0.28	1.01±0.19	2.35±0.21
**Solyc09g092130.2.1**	Sucrose phosphate synthase	-	1.98	3.45	-	0.42±0.17	1.99±0.18

b)

Gene ID	Descrizione	RNA-seq			qRT-PCR		
		V	M	MV	V	M	MV
**Solyc10g052470.1.1**	Myb family transcription factor	2.49	-2.62	-	2.41±0.19	-0.08±0.17	-
**Solyc12g100330.1.1**	chromomethylase 3	-1.39	-	-	-0.05±0.16	-	-
**Solyc02g094520.2.1**	Histone-lysine N-methyltransferase	-0.71	-	-	-0.31±0.18	-	-
**Solyc07g008240.2.1**	Non-symbiotic hemoglobin protein	-	-2.05	-2.86	-	-1.31±0.17	-1.89±0.09
**Solyc04g071800.2.1**	Cytochrome P450	1.18	-2.53	-1.11	1.70±0.19	-0.36±0.16	0.72±0.21
**Solyc05g005280.2.1**	Poly polymerase catalytic domain containing protein expressed	2.61	2.73	4.00	3.84±0.19	1.04±0.20	3.95±0.21
**Solyc10g085870.1.1**	UDP-glucosyltransferase family 1 protein	0.76	2.42	3.78	1.98±0.19	1.58±0.12	3.40±0.26
**Solyc09g092130.2.1**	Sucrose phosphate synthase	-	1.98	3.45	-	1.13±0.19	2.94±0.19

Table S9 Validation of mRNA-seq results by qRT-PCR; expression values are reported as the $\log_2$ of Fold Change in respect to C plants; (a) ubiquitin conjugating enzyme and (b) Elongation factor 1 were used as reference genes
